# Sleeping Birds Do Not Respond to Predator Odour

**DOI:** 10.1371/journal.pone.0027576

**Published:** 2011-11-16

**Authors:** Luisa Amo, Samuel P. Caro, Marcel E. Visser

**Affiliations:** Department of Animal Ecology, Netherlands Institute of Ecology, Wageningen, The Netherlands; University of Alberta, Canada

## Abstract

**Background:**

During sleep animals are relatively unresponsive and unaware of their environment, and therefore, more exposed to predation risk than alert and awake animals. This vulnerability might influence when, where and how animals sleep depending on the risk of predation perceived before going to sleep. Less clear is whether animals remain sensitive to predation cues when already asleep.

**Methodology/Principal Findings:**

We experimentally tested whether great tits are able to detect the chemical cues of a common nocturnal predator while sleeping. We predicted that birds exposed to the scent of a mammalian predator (mustelid) twice during the night would not go into torpor (which reduces their vigilance) and hence would not reduce their body temperature as much as control birds, exposed to the scent of another mammal that does not represent a danger for the birds (rabbit). As a consequence of the higher body temperature birds exposed to the scent of a predator are predicted to have a higher resting metabolic rate (RMR) and to lose more body mass. In the experiment, all birds decreased their body temperature during the night, but we did not find any influence of the treatment on body temperature, RMR, or body mass.

**Conclusions/Significance:**

Our results suggest that birds are not able to detect predator chemical cues while sleeping. As a consequence, antipredatory strategies taken before sleep, such as roosting sites inspection, may be crucial to cope with the vulnerability to predation risk while sleeping.

## Introduction

Despite being one of the most frequent behaviours of animals, sleep is also one of the less studied behaviours (see [1] for a review). Sleep involves energy saving [2], [3], as well as a restorative function for the immune system [4], and/or for the brain (e.g. memory consolidation [5], [6], [7]. However, sleep not only has benefits [8], but also has costs. The most important cost is probably the risk of predation. While asleep, an animal is less responsive to its environment, and may therefore fail to detect cues associated with the presence of a predator. Like any other behaviour, the balance between the benefits and costs may determine when, where and how animals sleep [1].

It has been shown that animals can change their circadian sleeping period to avoid the risk of predation. For example, free-living rats shifted from nocturnal to diurnal activity in response to human-associated changes in predator (Red Fox, *Vulpes vulpes*) activity [9]. Such shift in the sleeping period as a response to predation risks has also been suggested in birds. Several small-size species of Procellariiforms evolved toward nocturnal live despite a poor night vision, presumably to avoid predation by skuas (Stercorariidae) or gulls [10], [11]. Animals may also reduce predation risk by carefully selecting the places where to sleep [12], [13], [14]. This may be especially important for monophasic sleepers that tend to concentrate sleep into a single part of the day [15], [16]. Many bird species are monophasic sleepers and at high latitudes during winter, the time they spend sleeping is even longer than the daily period of activity. Birds that nest in cavities often also use these for roosting at night to avoid predation by owls, as roosting in cavities is usually safer than in tree canopies. For example, great tits that did not roost in nest boxes were more predated by owls, and therefore, had a lower overwinter survival than birds that were regularly observed sleeping in nest boxes [17].

Furthermore, when animals perceive some risk of predation before sleeping, they may also modify how they sleep. It has been shown that collared doves, *Streptopelia risoria*, exposed to the presence of a mustelid predator before sleeping increased the number of sleeping interruptions by opening their eyes to scan the environment [18]. Perceived increase in the risk of predation, not due to the real presence of a predator but to the absence of conspecifics (e.g. doves [18]), the vigilance levels of conspecifics (e.g. gulls [19]) or the distance to risky areas (e.g. mallards [20]) have also been shown to influence sleep patterns in birds. The position within a group leads also to differences in the perceived risk of predation that may be translated into differences in sleep patterns. For example, Rattenborg and collaborators [21], [22] found that when mallards were located on the edge of a group, and therefore, they perceived an increase in the risk of predation, they showed an increase in the proportion of time spent in unihemispheric sleep, compared to when they were safely sleeping surrounded by conspecifics [21], [22]. This unihemispheric sleep allows birds to sleep with one hemisphere and scan the environment with the other hemisphere at the same time.

Not only the position within a group in social animals but also other factors may increase the susceptibility of animals to the risk of predation while sleeping, by decreasing their ability to monitor the environment. For example, it is known that many bird and mammal species decrease their body temperature during cold nights in order to decrease the costs associated with the maintenance of a constant and elevated body temperature [23], [24]. By entering nocturnal hypothermia, and therefore, reducing their metabolic rate at night, birds can reduce starvation risk. However, this decrease in temperature also entails changes in the sleep pattern [25] that may cause a lower ability to detect an approaching predator while sleeping. Birds are known to be able to modify the degree of their body temperature decrease during the night in relation to the perceived risk of predation. For example, when pigeons were exposed during daytime to a model of a flying hawk, they did not decrease their body temperature as much as control pigeons during the following night [26]. This evidence suggests that animals can compensate for their loss of awareness while sleeping, not only by selecting safe places where to sleep but also by modifying their sleep patterns in relation to the perceived risk of predation before getting asleep. However, what happens once the animal is already sleeping? Can sleeping animals obtain information about the risk of predation like awake animals?

Many animals use the chemical cues of predators to ascertain their presence, especially under low visibility conditions [27]. Although evidence is still scarce in birds, recent experiments suggests that different bird species detect and use the chemical cues released by predators [28], [29]. For example, when blue tits, *Cyanistes caeruleus*, found predator chemical cues inside the cavity where they were feeding their nestlings, birds delayed their first entry in the nest-box. They perched on the hole of the nest-box and refused to enter more often when the nest-box contained predator odour than when it contained control scents. In those cases, they looked around and inside without entering [28]. Hole-nesting birds such as great *Parus major* and blue tits use cavities both for roosting in winter and for breeding in spring, where they can encounter predators such as mustelids. For an accurate assessment of the predation risk before entering a cavity, chemical cues are expected to be more efficient than visual cues, and they therefore may allow birds to avoid a risky encounter inside the cavity [28]. However, are chemical cues also useful for sleeping animals to detect an approaching predator during the night?

The aim of this study was to analyse whether birds are able to use their chemosensory abilities to detect predators while sleeping. We simulated a natural situation in which a bird is sleeping in a cavity and a predator approaches quietly, so that the bird can only perceive the chemical cues of the predator. As a measurement of how deeply a bird sleeps we used the Resting Metabolic Rate (RMR) and body temperature. The assumption is that a bird that is deeply sleeping consumes less oxygen than an awake and alert bird. We hypothesised that if sleeping birds are able to detect the chemical cues of predators, they would spend less time sleeping and arouse more often. We also predicted that under the risk of predation, birds would not decrease their body temperature as much as birds exposed to a control scent. And as a result of all this, we expected birds exposed to predator chemical cues to lose more weight than control birds and have a higher RMR. Alternatively, if sleeping birds, with reduced body temperature, are not able to detect the scent of predators, we would not expect differences in body temperature, RMR and body weight lost between birds exposed to the different treatments. As an experimental species we used the great tit, a species known to detect and use the chemical cues of mustelids to assess the risk of predation while selecting cavities to sleep [29]. We used mustelid urine as a cue for the presence of a predator. Mustelids are crepuscular and nocturnal hunters to which birds may be exposed while sleeping in cavities.

## Results

**Figure 1:**
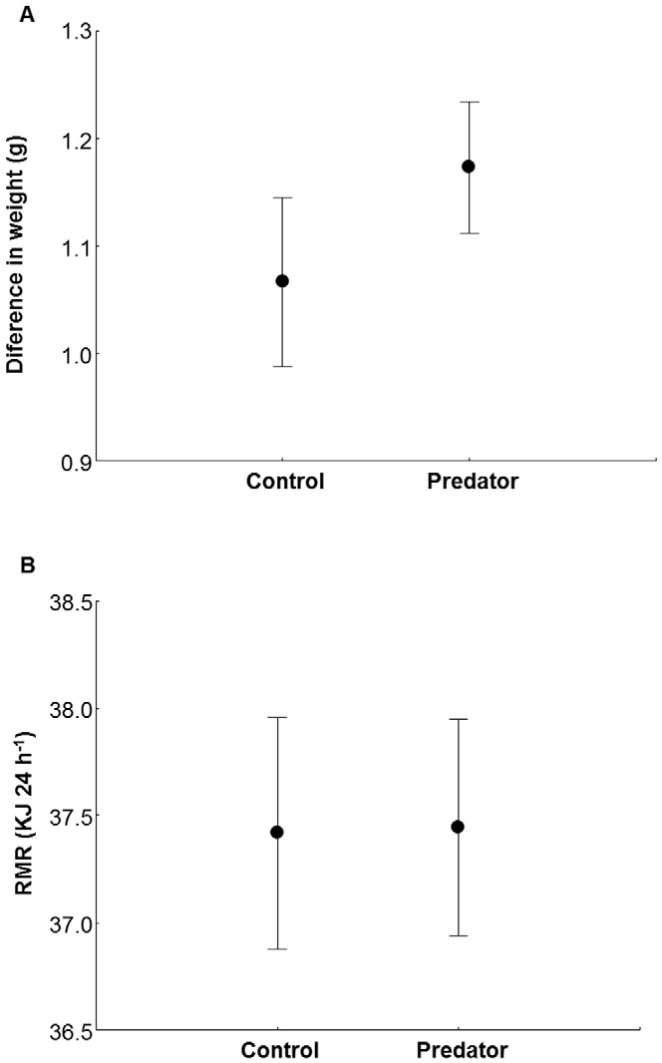
Weight loss and Resting Metabolic Rate. Mean ± SE **a**) Loss of weight (g) and **b**) Resting Metabolic Rate (KJ 24 h^−1^) of great tits exposed to predator scent or to control scent during the night in a respirometry chamber at 10°C.

**Figure 2:**
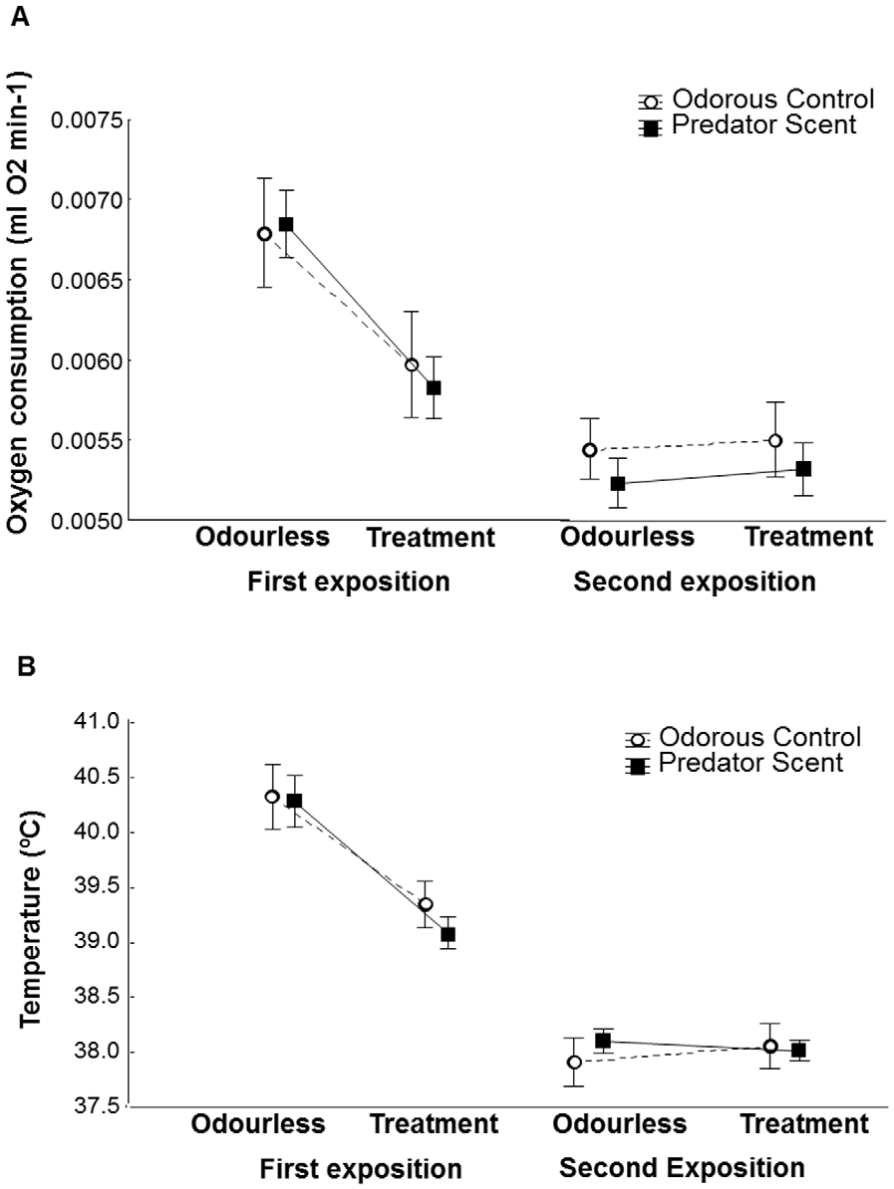
Oxygen Consumption and Body Temperature. Mean ± SE **a**) Oxygen consumption (ml O_2_ min^−1^) and **b**) Body temperature (°C) of great tits before (odourless control) and during the exposition to predator scent (treatment: predator (mustelid, full squares) or odorous control scent (rabbit; open circles) at the beginning of the sleeping period (first exposition, 30 minutes after being introduced in the respirometry chamber) and five hours later (second exposition).

There was no difference in the weight lost during the night between sexes or treatments (Table 1; Fig. 1a) and their interaction was also not significant (Table 1). RMR was related to the mean weight of the birds (Table 1), with heavier birds having higher RMR, but did not differ between sexes or treatments nor their interaction (Table 1; Fig. 1b). When exposed for the first time to the scents, birds were decreasing their oxygen consumption (F_1,23_ = 29, *p*<0.0001) but treatment did not affect this decrease (F_1,23_ = 0.02, *p* = 0.89) and the interaction between time and treatment was not significant (F_1,23_ = 0.34, *p* = 0.56; Fig. 2a). For the second scent exposition, there was no difference in the oxygen consumption before (in odourless condition) and during the scent exposition (F_1,28_ = 0.26, *p* = 0.61), nor between treatments (F_1,28_ = 0.77, *p* = 0.39). The interaction between time and treatment was not significant (F_1,28_ = 0.01, *p* = 0.93; Fig. 2a).

**Table 1:** Statistics.

Body weight loss		
	Sex:	F_1,26_ = 0.16, *p* = 0.69
	Treatment:	F_1,26_ = 0.88, *p* = 0.36
	Sex*Treatment:	F_1,26_ = 0.88, *p* = 0.36

RMR		
	Body weight:	F_1,25_ = 6.74, *p* = 0.02
	Sex:	F_1,25_ = 0.13, *p* = 0.72
	Treatment:	F_1,25_ = 0.16, *p* = 0.70
	Sex*Treatment:	F_1,25_ = 1.18, *p* = 0.29

Body temperature		
	Body weight:	F_1,23_ = 1.05, *p* = 0.32
	Sex:	F_1,23_ = 0.98, *p* = 0.33
	Treatment:	F_1,23_ = 0.003, *p* = 0.95
	Sex*Treatment:	F_1,14_ = 1.03, *p* = 0.32

Statistics and significance levels of loss of body weight, resting metabolic rate, and mean body temperature of male and female great tits exposed two times during the night to the scent of a predatory mammal (mustelid) or a non predatory mammal (rabbit) in a respirometry chamber at 10°C.

The mean body temperature of birds during the night was not related to the mean weight of the birds and did not differ between sexes or treatments (Table 1), and the interaction between sex and treatment was also not significant (Table 1). When analysing in detail the body temperature of birds before (in odourless condition) and during the scent exposition, results show similar patterns than those obtained for the oxygen consumption. The body temperature of birds was decreasing while they were exposed to the scent for the first time (F_1,26_ = 67.47, *p*<0.0001), but this was not modulated by the treatment to which birds were exposed (F_1,26_ = 0.27, *p* = 0.60) and the interaction between time and treatment was not significant (F_1,26_ = 0.70, *p* = 0.41; Fig. 2b). In the second exposition to the scent, the body temperature of birds neither differ before (in odourless condition) and during the exposition (F_1,26_ = 0.19, *p* = 0.67) nor between treatments (F_1,26_ = 0.11, *p* = 0.74). The interaction between time and treatment was not significant (F_1,26_ = 2.48, *p* = 0.13; Fig. 2b).

## Discussion

Our results suggest that sleeping birds are not able to detect the chemical cues of predators, as they did not exhibit any response to the predator treatment in the physiological variables that we measured here. Birds exposed to predators' scent did not exhibit higher metabolic rates, higher temperatures or a greater loss of body weight compared to birds exposed to the scent of a non-predatory mammal. Birds in our experiment decreased their body temperature by 3.5°C, meaning that they only had a moderate degree of nocturnal hypothermia. They did not enter into torpor, which can be defined as a drastic decrease of body temperature reaching values of more than 12°C [23]. However, despite this low decrease in temperature they did not react to the chemical cues of predators. An alternative explanation could be that birds responded in a similar way to the scent of a non-predatory mammal than to a predatory mammal. However, awake birds are known to exhibit behavioural responses to predator scent but not to other scents (including rabbit scent [30]) or odourless controls [28], [30]. Thus, if great tits are physiologically able to discriminate between a predator scent and other scents while they are awake [29], we would also expect them to discriminate between such scents while asleep. Although a differential discrimination capacity while awake or asleep might seem unlikely, we would have needed a third group of birds that would not have been exposed to any organic scent to formally exclude this possibility. This would have allowed us to know whether the physiological profiles observed early in the night reflect the normal nocturnal variation in oxygen consumption and body temperature or a general, non-specific, response to odors. Further research is needed to examine whether the ability to discriminate scents changes during the sleeping period. Another explanation could be that the lack of difference between treatments is a methodological artefact due to the stress of manipulation that may have caused birds to remain alert during the night. However, since birds decreased their body temperature during the first minutes of the experiment (see Fig. 2b), this explanation seems unlikely. Therefore, our results suggest that birds are not able to detect chemical cues of predators while they are sleeping, or at least they do not respond to them. Despite the fact that chemical cues may provide a first warning of the presence of a predator, they often persist after the predator has left the area [27]. Therefore, other cues, such as auditory or vibratory cues may reveal the current presence of the predator more accurately than chemical ones. So, such cues may probably be more meaningful stimuli for eliciting an arousal response.

To compensate for their susceptibility to the risk of predation while sleeping, birds may exhibit antipredatory strategies prior to sleep. One of the main strategies is to select safe places where to sleep. It has been shown that birds actively select for cavities before dusk [31], and that they avoid cavities containing signals of predators as well as predation. For example, great tits avoid roosting in cavities containing traces of predator presence, i.e. mammal fur and mangled feathers [32]. Results of a previous study have also shown that when great tits were offered two nest-boxes for roosting, with one of them containing the scent of a predator, significantly more birds slept outside of any nest-box compared to situations where one of the nest-boxes contained control scent [29]. Therefore, the use of chemical cues of predators seems to have an important role in selecting safe roosting places. Furthermore, predation risk may also have influenced population differences in the use of roosting places for sleeping. For example, blue tits from populations where mustelid predation is high do not roost in nest-boxes, while blue tits from populations where owl predation is prevalent do [33].

In conclusion, our results demonstrated that birds do not detect predator chemical cues while sleeping, or at least, that they lose their capacity to discriminate between chemical cues emitted by predators and other scents. Therefore, previous antipredatory strategies, such as roosting sites inspection, may be crucial in determining their night survival. Further studies examining in detail sleep composition (e.g. percentages of time in REM or SWS sleep) as well as type of sleep (bi- or unihemispheric sleep) are needed to study the ability of birds to detect chemical cues during the sleep period in more detail. Such techniques may reveal encephalic responses [34] to scents that may not affect the physiological measurements considered in our study, as previous evidence suggests that sleeping animals can detect scents [35] even if they do not always respond to them, i.e., they do not wake up [36].

## Materials and Methods

### Study species

The experiment was performed with 29 captive great tits (12 males and 17 females) in March 2008. Birds were hand-reared and therefore, used to be handled. Birds were housed individually in cages (0.9×0.5×0.4 m), with wooden bottom, top, side, and rear walls, a wire-mesh front, and three perches. The bottom was covered with wood chips. The birds were kept in three rooms under ambient temperature (outside temperature during the experiment: maximum 6°C during the day and minimum −2°C during the night) and natural winter daylight artificially supplemented with fluorescent light tubes under natural photoperiod. They were provided with *ad libitum* water, sunflower seeds, a commercial dry mixture (proteins, trace elements, minerals, and vitamins), a fresh mixture of raw heart and live mealworms. Birds were deprived of food 90 min before the start of the experiment in order to stimulate them to enter into torpor, to mimic natural conditions. Birds were introduced in the respirometry chamber at sunset (6:00 pm) and they were removed and returned to their cages at 8:00 am the next morning.

### Experimental design

#### Treatments

**Figure 3:**
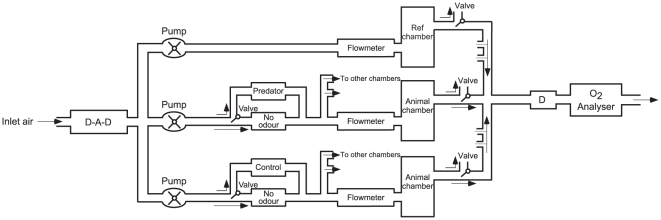
Respirometer Schema. Schematic representation of the respirometer used in the experiment. From left to right: carbon dioxide and humidity are removed from inlet air thanks to a column containing one layer of Ascarite (A) surrounded by two layers of Drierite (D). Pumps force the air in three different circuits: i) a reference circuit (top) containing an empty chamber used to calculate the amount of oxygen used by the birds in the other chambers, ii) a second circuit (middle) leading to the three chambers containing the birds exposed to predator (ferret) odour, and iii) a third circuit (bottom) leading to the three chambers containing birds exposed to the control (rabbit) odour. In each of these two last circuits, a first valve lead the inlet air either in a compartment containing the urine-damped filter paper, or a compartment containing a dry, odourless, filter paper. A flowmeter setting the flow rate to 250 ml min^−1^ precedes each animal chamber. A second set of valves, installed after each animal chamber, controls from which chamber the oxygen concentration is measured.

Birds were randomly assigned to one of the two treatment groups: an experimental group exposed to the odour of a potential mammal predator (fresh ferret urine), and a control group exposed to the odour of an herbivorous mammal (fresh rabbit urine). We added an absorbent paper soiled with the correspondent odour to a box situated ahead of each respirometry chambers (Fig. 3). Each test day, we used new papers and cleaned the respirometry chambers.

We obtained predator odour by placing clean absorbent papers under the cages of five male ferrets (*Mustela furo* L.). We used ferrets because, even though ferrets are not natural predators of great tits, the scent (especially the one produced by anal sac secretion that they use to mark the territory) is very similar to those of other mustelids, such as *M. erminea* and *M. putorius* [37] that include birds in their diets. Ferret scent has been used in several vertebrates that are not depredated by this mustelid, including great tits, and it induced avoidance-associated behaviours (e.g. [28], [29], [38]). We chose papers soiled with fresh urine and gland secretions associated to scent-marking behaviour, whereas papers containing faeces were discarded. We placed papers under the ferret cages three days before the experiment, to ensure odour collection. When collecting papers daily for the experiment, we selected wet papers containing recent cues. This method of odour collection has proven successful in previous studies with hole breeding passerines [28], [29].

The control treatment was obtained by using the same methodology to collect rabbit (*Oryctolagus cuniculus* L.) urine. We used this odorant control because rabbits are herbivorous and therefore, their chemical cues should not be associated with a predation risk for the birds.

#### Body temperature

At least 72 hours before the start of the experiment, birds were anaesthetized under isoflurane (Forene, Abbott b.v., Hoofddorp, The Netherlands) and a body temperature transponder (IPTT-300, Plexx, Elst, The Netherlands) was implanted under the skin of their back. Body temperature was recorded every 30 min during the first 5 hours of each night's experiment, using a portable reader (DAS-6007, Plexx, Elst, The Netherlands). Two transponders did not work on the day of the experiment, so these birds were removed from the temperature analysis.

#### RMR measurements

We measured Resting Metabolic Rate (RMR) in terms of oxygen consumption, during 5 consecutive nights (5 or 6 birds per night), in an open-circuit respirometer (see [39]; Fig. 3). Birds were weighted and isolated in 6 sealed respirometer chambers (0,76 l) and placed in the darkness of a climate cabinet (Sanyo MIR-553, Sanyo E&E Europe BV, Etten-Leur, The Netherlands) at 10°C to induce birds to enter hypothermia. Chambers 1 to 3 were allocated to predator odour, chambers 4 to 6 to control odour. Each morning, the chambers were successively cleaned using dish soap and methanol to remove persistent odours. After the third night, all the chambers and the tubing between the odour source and the chambers were replaced. H_2_O and CO_2_ were removed from the inlet air (blown into the animal chamber) respectively with Drierite® (6 mesh, Sigma-Aldrich Chemie b.v., Zwijndrecht, The Netherlands) and Ascarite® (5–20 mesh, Fluka, Zwijndrecht, The Netherlands). Air flow rate was set to 250 ml min^−1^ with flowmeters (Brooks Instrument b.v., Ede, The Netherlands) previously calibrated using a soap bubble method (Bubble-O-Meter, LLC, Dublin, OH, USA). Oxygen content of outlet air was measured with an oxygen analyzer (Servomex 4100, Servomex BV, Zoetemeer, The Netherlands). Oxygen consumption (ml O_2_ min^−1^) was calculated as the difference in oxygen concentration between air from the respirometer chambers and reference air from an empty chamber (Fig. 3). As only one oxygen analyzer was used, measurements alternated between the six experimental, plus one reference, chambers every 5 min. The oxygen consumption was converted to metabolic rate (kJ 24 h^−1^) by assuming an energetic equivalence of 20 kJ per liter of O_2_.

During each night, birds were exposed to two 30 minute periods of odour (half an hour after the birds have been settled in the chamber, and five hours later). The rest of the time, they were exposed to fresh inlet air (see “No odour” compartment on Fig. 3). In that way, we simulated two approaches of a mustelid predator at the beginning and in the middle of the sleep period. Furthermore, with this experimental design, we obtained the body temperature and oxygen consumption measurements of each bird under two conditions: odorless air (odorless control) and scented air (treatment: predator or odorous control).

### Statistical Analysis

The weight of birds before the experiment did not differ between treatments (F_1,26_ = 0.17, *p* = 0.69), but differed between sexes (ANOVA, F_1,26_ = 5.37, *p* = 0.03), and the interaction between treatment and sex was not significant (F_1,26_ = 1.34, *p* = 0.26).

We used two-way Analysis of Variance (ANOVA) to analyse the differences between treatments and sexes in the weight lost by birds. We used two-way Analysis of Covariance (ANCOVA) to examine the differences in the Resting Metabolic Rate (RMR) and in the mean body temperature in relation to the treatment and the sex, including the mean weight (the mean between the initial and final body weight) as a covariate. We included the interaction between treatment and sex in all the models. Differences between treatments (between subject factor) in the oxygen consumption and in the body temperature of birds before (odourless condition) and during the scent exposition (time, within subject factor) were analysed separately for the first and second periods of scent exposition by using repeated measure ANOVA. We included the interaction between treatment and time during exposition to test whether there were differences between treatments in the response of birds before (odourless condition) and during each scent exposition.

### Ethics statement

Birds were healthy during the study and they did not exhibit any sign of stress due to the implantation or removal of transponders or to the experiment. They resumed their normal behaviour immediately after they returned to their cages after implantation and removal of transponders and after being tested in the respirometry chamber. This experiment was carried out under license of the Animal Experimental Committee of the KNAW (DEC protocol no CTE 0701).
